# Editorial: Diet-microbe-host interactions in metabolic syndrome

**DOI:** 10.3389/fnut.2023.1129200

**Published:** 2023-01-18

**Authors:** Gratiela Gradisteanu Pircalabioru, Manfredi Rizzo, Anca Pantea Stoian, Mariana Carmen Chifiriuc

**Affiliations:** ^1^Research Institute of University of Bucharest (ICUB), Bucharest, Romania; ^2^Academy of Romanian Scientists, Bucharest, Romania; ^3^PROMISE Department, University of Palermo, Palermo, Italy; ^4^Department of Diabetes, Nutrition and Metabolic Diseases, Carol Davila University of Medicine and Pharmacy, Bucharest, Romania; ^5^Romanian Academy, Bucharest, Romania; ^6^Faculty of Biology, University of Bucharest, Bucharest, Romania

**Keywords:** microbiota, metabolic syndrome, diet, obesity, nutrition

Affecting around 25% of the world's adult population, metabolic syndrome (MetS) is a cluster of metabolic disorders such as insulin resistance, dysglycemia, dyslipidemia, hypertension and central adiposity with visceral fat accumulation (1, 2). Given its impact on host immunity and metabolism, the gut microbiome is a possible culprit in triggering MetS (3, 4). The scope of the “*Diet-microbe-host interactions in metabolic syndrome*” Research Topic was to report new findings in the field of MetS including microbiota patterns linked to metabolic disease, effects of dietary/probiotic/pre-biotic/post-biotic interventions on MetS complications and inflammatory status, host-microbe interactions in metabolic disease, clinical trials using microbiota targeting, as well as personalized medicine approaches.

In this special e-collection there are 11 papers covering several of the above-mentioned aspects.

Reports of gut dysbiosis associated with Western diet consumption have led to increased interest in the role of the microbiome in the development of obesity and MetS (5). To better study these aspects, it is crucial to develop research models that accurately reflect MetS affected populations. Chehade et al. have formulated a modified Standard American Diet (mSAD) to induce the physiological parameters associated with MetS in mice (*A modified standard American diet induces physiological parameters associated with metabolic syndrome in C57BL/6J mice*). The mSAD administration was associated with altered glucose metabolism, increased body weight and reduced abundance of the *Akkermansia* genus.

Chronic systemic inflammation had long been considered as a major player in the development and progression of non-communicable diseases such as MetS (6). Pro-inflammatory cytokines, other adipokines, along free fatty acids secreted from adipose tissue may activate and/or accelerate the development of insulin resistance and other pathological effects. Dietary manipulations may improve the inflammatory status and the microbiota profile in obesity. A study performed on C57BL/6 mice studied the effects of habitual consumption of the traditional peanut cultivar, HN, and the new high-oleic peanut cultivar, HO, as an integral part of diet-induced obesity (*Metabolic and microbiome alterations following the enrichment of a high-fat diet with high oleic acid peanuts versus the traditional peanuts cultivar in mice*) (Anavi-Cohen et al.). The new high oleic acid cultivar was metabolically superior to the traditional peanut type and was associated with a better inflammatory state and microbiome signature (lower levels of the *Erysipelotrichaceae* family).

In mice, acidic activated charcoal improved high fat die-induced obesity and insulin resistance in a dose-dependent manner without any serious adverse effects. Metabolomic analysis of cecal contents revealed that cholesterol, neutral lipids, and bile acids were excreted at significantly higher levels in the feces of mice under charcoal treatment (*Acidic activated charcoal prevents obesity and insulin resistance in high-fat diet-fed mice*) (Zhang et al.).

Using an animal model for non-alcoholic fatty liver disease (NAFLD), Jian et al. showed that long-term exposure to excessive dietary valine influenced amino acid and fatty acid metabolism by promoting fatty acid synthesis, impairing amino acid metabolism, and inducing amino acid imbalance (*Amino acid and fatty acid metabolism disorders trigger oxidative stress and inflammatory response in excessive dietary valine-induced NAFLD of laying hens*).

Another nutritional supplement, represented by astaxanthin (ATX), a xanthophyll carotenoid, was reported to prevent steatohepatitis and hepatic oxidative stress in mice with diet-induced obesity (*Astaxanthin from Haematococcus pluvialis prevents high-fat diet-induced hepatic steatosis and oxidative stress in mice by gut-liver axis modulating properties*) (Wang, Xu et al.). ATX exerted important effects on the microbiome by significantly inhibiting the growth of obesity-related genera *Parabacteroides* and *Desulfovibrio* while promoting the growth of *Akkermansia* and *Allobaculum* genera.

Polydatin (POD), a natural precursor and glycosylated form of resveratrol had important effects on the microbiome by increasing the abundance of beneficial bacterial genera (*Butyrycimonas, Bifidobacterium*). POD administration altered the glucolipid dysmetabolism, insulin resistance, and non-alcoholic fatty liver disease by reducing the oxidative stress and preventing AMP-activated protein kinase (AMPK) suppression induced by high-fructose diet in mice (*Polydatin, a glycoside of resveratrol, is better than resveratrol in alleviating non-alcoholic fatty liver disease in mice fed a high-fructose diet*) (Zhao et al.).

Treatment with the polyphenol rich Black chokeberry (BCP) reduced body weight, liver, and white adipose tissue weight and alleviated hepatic steatosis and dyslipidemia in rats with obesity [*Modulation of the gut microbiota and lipidomic profiles by black chokeberry (Aronia melanocarpa L.) polyphenols via the glycerophospholipid metabolism signaling pathway*] (Zhu et al.). BCPs supplementation increased the relative abundance of genera such as *Bacteroides, Romboutsia, Prevotella*, and *Akkermansia* and decreased the relative abundance of genera *Clostridium* and *Desulfovibrio*.

Short-chain fatty acids (SCFA) such as acetate, propionate and butyrate are produced by bacterial fermentation of non-digestible carbohydrates. HFD (high fat diet) before and during pregnancy significantly induced obesity and worsen glucose tolerance, lipid metabolism and insulin sensitivity in gestational mice. Ding et al. emphasized the importance of the rhythmicity of gut microbiota-derived SCFAs in mediating circadian disruption in response to the HFD in gestational mice (*A high-fat diet disrupts the hepatic and adipose circadian rhythms and modulates the diurnal rhythm of gut microbiota-derived short-chain fatty acids in gestational mice*).

In human studies, anti-inflammatory agents together with dietary interventions may be therapeutically useful in treating and preventing MetS (7, 8). A meta-analysis performed by Wang, Liu et al. (*Effects of dietary intervention on inflammatory markers in metabolic syndrome: A systematic review and meta-analysis*) provided evidence that dietary intervention could improve immunological properties, particularly IL-6, in MetS. Subsequent analysis based on subgroups indicated that these results were affected by dietary patterns in MetS. Despite this, it did not reveal the association between dietary intervention and the IL-1β, CRP, and TNF-α levels. Nevertheless, more research is needed to underpin the mechanisms underlying the effect of dietary intervention on MetS associated inflammatory markers.

In women with obesity, a low-glycemic diet significantly altered the microbiota by lowering the abundance of G*emmiger formicilis, Collinsella aerofaciens*, and *Escherichia coli* (*Beneficial effects of a low-glycemic diet on serum metabolites and gut microbiota in obese women with Prevotella and Bacteriodes enterotypes: A randomized clinical trial*) (Hur et al.). Importantly, the low glycemic diet was more efficient in women with obesity and a *Prevotella* dominant enterotype suggesting that dietary patterns could impact metabolic traits differently in different enterotypes, indicating the need of a personalized diet based on enterotypes. Further studies are needed to find appropriate diets for each enterotype.

The use of microbiome regulation (especially the enrichment of some SCFA-producing bacteria or the targeting of specific SCFA-producing bacteria, such as the representative *A. muciniphila* strain, to improve the intestinal environment to help adults with obesity lose weight) has become an attractive strategy reported in several clinical studies (*The therapeutic effect of SCFA-mediated regulation of the intestinal environment on obesity*) (You et al.). Nevertheless, the microbiota is a complex ecosystem, and whether SCFA play a role directly or by regulating gut microbes remains to be explored.

Dietary interventions are essential tools for modifying the microbiota and improve host health. Future studies characterizing the effects of dietary components through the host-microbe axis are pivotal to provide evidence-based dietary interventions to prevent and ameliorate MetS and other metabolic disorders.

## Data availability statement

The original contributions presented in the study are included in the article/supplementary material, further inquiries can be directed to the corresponding author.

## Author contributions

GG: draft preparation. AP, MR, and MC: review and editing. All authors contributed to the article and approved the submitted version.
